# Formation of C_1_ oxygenates by Activation of Methane on B, N Co‐Doped Graphene Surface Decorated by Oxygen Pre‐Covered Ir_13_ Cluster: A First Principles Study

**DOI:** 10.1002/open.202400287

**Published:** 2025-02-11

**Authors:** Jemal Yimer Damte, Jiri Houska

**Affiliations:** ^1^ Department of Physics and NTIS – European Centre of Excellence University of West Bohemia in Pilsen Univerzitni 8 30614 Plzen Czech Republic

**Keywords:** Oxidative coupling of methane, Methane activation, BNG-Ir_13_O cluster, Adsorption, Dehydrogenation

## Abstract

The environmentally friendly conversion of methane to value‐added chemicals is studied by ab‐initio calculations. We focus on the adsorption and dehydrogenation of methane were investigated on Ir13 cluster supported by boron nitrogen co‐doped graphene (BNG). We show that the BNG‐Ir_13_ cluster with low oxygen coverage exhibits more negative adsorption energy of methane (−0.44 eV) and lower activation energy barrier for its dissociation (1.24 eV for the second dehydrogenation step) compared to the cluster with high oxygen coverage. Next, assuming abundant CH_3_ and CH_2_ species due to a proper temperature control preventing further dehydrogenation, we study competitive C−O coupling reactions leading to the formation of value‐added chemicals. The activation energy barriers for the formation of methanol and formaldehyde are on BNG‐Ir_13_ are once again lower at a lower oxygen coverage. Furthermore, we study hydrogen recombination and confirm that H_2_ molecules can be formed on these surfaces. Based on these findings, the low‐oxygen‐coverage BNG‐Ir_13_ cluster emerges as a promising catalyst for the selective conversion of methane to methanol and formaldehyde, as well as for hydrogen production.

## Graphical abstract

We demonstrate that the BNG‐Ir13 cluster with low oxygen coverage exhibits a more negative methane adsorption energy and a lower activation energy barrier for methane dissociation compared to its high‐oxygen‐coverage counterpart. This suggests that the low‐oxygen‐coverage BNG‐Ir13 cluster is a promising catalyst for the selective conversion of methane to valuable products such as methanol and formaldehyde, as well as for efficient hydrogen production.

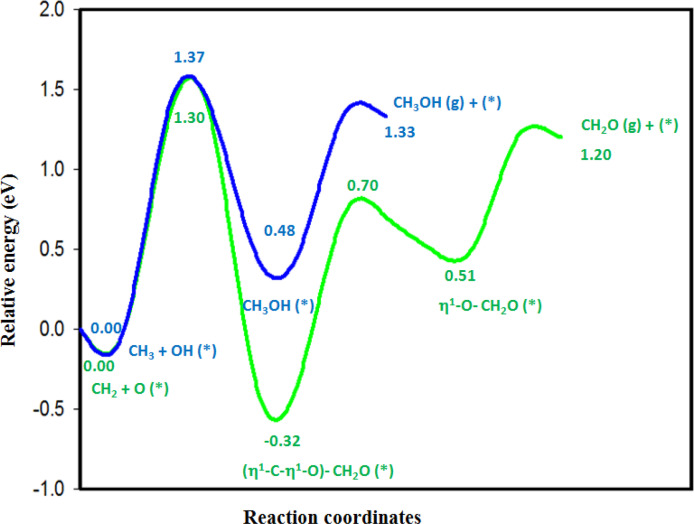

## 1 Introduction

Natural gas predominantly consists of methane, which is extensively utilized as a primary fuel. The utilization of methane has gained considerable interest in recent times due to the depletion of fossil fuel reserves and the requirement for innovative technologies. However, the emission of methane directly or via oxidation to CO_2_, contributes to the issue of global warming. Therefore, methane conversion is an attractive alternative to maintaining environmental safety and producing valuable chemicals.[^1^, ^2^, ^3^, ^4^] The conversion of methane to value‐added chemicals can occur through either an indirect or direct route. The indirect route involves multiple steps, producing synthesis gas intermediates via steam reforming. In contrast, the direct route is a single‐step process where methane reacts with an oxidant to directly yield the desired product. The prevalent approach for transforming methane into various value‐added chemicals involves an indirect process known as synthesis gas production. The synthesized gas is subsequently utilized to produce essential commodities via the Fischer‐Tropsch process. Even though synthesis gas production is a proven technology for methane conversions, it is the most cost‐intensive and multi‐step process.[^5^, ^6^, ^7^, ^8^] Hence, considerable literature has been published to find the most attractive routes in converting methane to value‐added products. The direct selective oxidation of methane to C_1_ oxygenates (methanol and formaldehyde) can be essential in addressing the cost issue by removing the synthesis gas production.
(1)

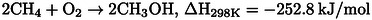



(2)

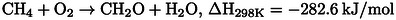




The process of converting methane to methanol and formaldehyde (reactions 1 and 2) is thermodynamically favorable, making the oxidative transformation of methane with oxygen an attractive method for methane conversion. These products are commonly used as feedstock in the petrochemical industry and can be easily transported at a low cost. However, achieving selective oxidation of methane has been a topic of intense debate in the scientific community. The direct conversion of methane to C_1_ oxygenates is particularly challenging due to the high reactivity of the desired product, which often leads to further oxidation to carbon dioxide, resulting in reduced product selectivity.[^9^, ^10^, ^11^, ^12^, ^13^, ^14^]

Over the last few decades, numerous studies have yielded significant insights into different catalysts for the oxidation of methane to C_1_ oxygenates. Transition metal oxides have shown promise as effective catalysts for this process. Both theoretical and experimental investigations have been conducted to explore their exceptional catalytic activity in selectively converting methane to valuable chemicals.[^15^, ^16^, ^17^, ^18^, ^19^, ^20^] For example, selective oxidation of methane to methanol has been reported using various catalyst at high‐temperature conditions.^[21]^ Due to the stability of methane, this catalyst operates at high temperatures to cleave the initial C−H bond of methane and produce free CH_3_ radicals on the surface. Under such conditions, the CH_3_ radical couples with surface oxygen to produce C_1_ oxygenates.[^22^, ^23^] However, this oxidant, at high‐temperature conditions, facilitates the over‐oxidation process and overwhelms the selective oxidation of methane to C_1_ oxygenates. Even though alternative oxidants are used for the selective oxidation of methane and can minimize the over‐oxidation process, the performance is deficient compared to oxygen. Methane is a stable molecule, requiring an efficient catalyst for its activation and conversion into various value‐added chemicals. Therefore, identifying an optimal catalyst is crucial. The catalyst used in this work activates methane under low‐temperature conditions and facilitates coupling reactions to produce C_1_ oxygenates, which can serve as raw materials for industrial applications.[^24^, ^25^]

Numerous studies have demonstrated that metal clusters are highly effective catalysts with desirable characteristics for various reactions. Transition metal clusters are recognized for their efficiency and importance in industrial applications. Among these, iridium (Ir) clusters stand out as versatile catalysts, playing a pivotal role in hydrocarbon reactions by efficiently activating C−C, C−N, and C−H bonds.[^26^, ^27^, ^28^, ^29^, ^30^] For instance, the activation of methane at low temperatures has been investigated on the IrO_2_ (110) surface.^[31]^ However, none of the available papers on Ir clusters supported by boron nitrogen co‐doped graphene deals with the conversion of methane to the C_1_ oxygenates.

Note that when the expensive iridium is used in minimal quantities, such as in clusters or nanoparticles, its catalytic activity can be significantly enhanced by supports like graphene (with possible dopants such as boron and nitrogen). This synergistic interaction not only improves performance but also reduces the required amount of Ir, thereby minimizing costs. Furthermore, iridium clusters supported on metal oxide surfaces or doped graphene have exhibited efficient catalytic activity for alkane dehydrogenation and hold great promise as catalysts for C−H bond activation.[^32^, ^33^, ^34^, ^35^, ^36^] Boron and nitrogen co‐doped graphene (BNG) exhibits improved anchoring of metal nanoparticles or clusters, preventing aggregation (however, this design is not to be cofused with using BNG itself as a catalyst, without metal clusters).

Based on this, we theoretically investigate the methane activation and the C−O coupling reactions on boron nitrogen co‐doped graphene decorated by oxygen pre‐covered iridium cluster (BNG‐Ir_13_O cluster). This work aims to convert methane to C_1_ oxygenates using an oxidant on boron nitrogen co‐doped graphene decorated by iridium cluster under mild temperature conditions.

## Computational Details

All calculations were performed in the framework of density functional theory (DFT) with the program package Vienna ab initio simulation package (VASP).^[37]^ The exchange‐correlation energy was calculated within the generalized gradient approximation (GGA) of the Perdew–Burke–Ernzerhof (PBE) functional,^[38]^ and the electron core interaction was described by the projector augmented wave (PAW) method.^[39]^ The Van der Waals correction proposed by Girmme DFT+D3 was applied to expand the description of the Perdew–Burke–Ernzerhof (PBE) functional and inter‐molecular dispersion interactions that were corrected.^[40]^ A plane wave basis set with a kinetic energy of 400 eV was used, and the reciprocal space was sampled with a 3×3×1 Monkhorst–Pack k‐point grid.^[41]^ The boron nitrogen co‐doped graphene surface constituted a periodic 6×6 super‐cell unit‐cell (12 N+12 B+48 C atoms) with the boron and nitrogen dopants positioned in the ortho configuration. A 15 Å vacuum thickness was used to separate the surface from its periodic images along the z direction. All atoms were allowed to relax in optimized geometry calculations, and spin polarization was considered.

To validate our calculations, we performed a benchmark analysis of Ir adsorption energies using a range of cutoff energies and k‐point settings. Based on that, the estimated uncertainty of the presented energies is 0.01 eV. The optimized lattice constant of bulk graphene was 2.46 Å, i.e. the same as experimental value. Additionally, various optimized geometries of the Ir_13_ cluster decorated on the boron‐ and nitrogen‐codoped graphene (BNG‐Ir_13_ cluster) were evaluated. The possible structures and their calculated energetics are presented in Figure S1 and Table S1, respectively.

The adsorption energy of the intermediates was calculated using the following definition:
(1)

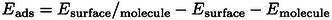




Where *E_surface_/_molecule_* is the total energy of surface together with a molecule, *E_surface_* is the energy of a clean surface and *E_molecule_* is the calculated energy of a molecule. The Climbing Image Nudged Elastic Band (CI‐NEB) method^[42]^ was applied to calculate the transition state of various elementary steps involved in methane dissociation on boron nitrogen co‐doped graphene decorated by Ir_13_ (BNG‐Ir_13_) cluster. The transition state corresponds to the highest energy along the reaction coordinate defined by NEB calculation. Vibrational frequencies were calculated for the optimized geometry intermediates and transition state structure; furthermore, zero‐point energy correction was included in the reaction energetics, which is calculated as follows:

(2) $$ZPE=\;\sum \frac{1}{2}hv,\;$$

**Figure 1:**
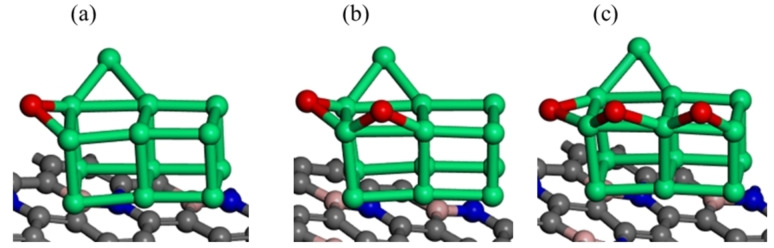
The most stable optimized structures of oxygen pre‐covered Ir_13_ cluster decorated on boron nitrogen co‐doped graphene surface (a) BNG‐Ir_13_O cluster (b) BNG‐Ir_13_O_2_ cluster and (c) BNG‐Ir_13_O_3_ cluster, atomic spheres: green, Ir; gray, C; deep blue, N; pink B; red, O.

Where h is Planck's constant and ν is the vibration frequency. Possible optimized geometries of Ir_13_ cluster on boron nitrogen co‐doped graphene surface were considered. The most stable oxygen pre‐covered BNG‐Ir_13_ cluster is shown below in Figure 1.

## 2 Results and Discussion

### 2.1 Oxygen pre‐Covered BNG‐Ir_13_ Cluster

Molecular oxygen is frequently used as an oxidant for the oxidative coupling of methane. However, due to the high energy barrier for the dissociation of oxygen molecules on the BNG‐Ir_13_ cluster, this study utilizes water adsorption and dissociation to obtain oxygen atoms. Various adsorption sites were analyzed for the adsorption of water onto the BNG‐Ir_13_ cluster, revealing that water adsorbs perpendicularly to the surface, with the oxygen binding to the top site of Ir in the cluster. The most stable adsorption energy observed for water on this surface was −0.78 eV, which exceeds previously reported values.[^43^, ^44^, ^45^] The optimized geometry of the adsorption of water on BNG‐Ir_13_ cluster is shown in the first panel of Figure S2 in the supporting information. Following the identification of stable water adsorption on the BNG‐Ir_13_ cluster, we investigated the sequential dehydrogenation of water to generate oxygen atoms. The optimized geometry of the initial, transition, and final states of water dehydrogenation on the cluster are illustrated in Figure S2 of the supporting information. The initial dehydrogenation of adsorbed water is the most facile step and exothermic in nature (as shown in Table S2). However, the second dehydrogenation step, which involves dehydrogenating OH to O+H to produce oxygen atoms on the BNG‐Ir_13_ cluster, is the rate‐limiting step and endothermic with a higher activation energy barrier. Consequently, a disproportionate reaction, 2OH→O+H_2_O, was considered on the BNG‐Ir_13_ cluster, resulting in the formation of oxygen atoms with a lower activation energy barrier (as indicated in Table S2), albeit with slightly endothermic reaction energy. Upon introducing the oxygen atom to the BNG‐Ir_13_ cluster, it was observed that the oxygen atom favored adsorption at the bridge site of Ir in the cluster (as depicted in Figure 1). During dehydrogenation, the hydrogen atom remained on the BNG‐Ir_13_ cluster, and we subsequently studied hydrogen recombination to form a hydrogen molecule. The result confirms that the formation of the hydrogen molecule is possible in oxygen pre‐covered BNG‐Ir_13_ cluster. The optimized structures of the initial states, the transition states, and the final states are presented in Figure S3 in the supporting information.

### 2.2 Adsorption of Methane and its Intermediates on Oxygen pre‐Covered BNG‐Ir_13_ Cluster

To examine the impact of oxygen coverage on methane dehydrogenations and the potential for C−O coupling reactions, we investigated the low and high oxygen coverage on the BNGIr_13_ cluster. This involved studying the adsorption of intermediates by progressively introducing oxygen to the BNG‐Ir_13_ cluster. We explored potential adsorption sites of oxygen atoms on the cluster and identified the bridge site as the most stable configuration independently of the coverage. To optimize the structure of the BNG‐Ir_13_O cluster, an oxygen atom was added to the BNG‐Ir_13_ cluster. Then, we repeated this process to obtain the optimized geometry of the BNGIr_13_O_2_ and BNGIr_13_O_3_ clusters, respectively.

**Figure 2:**
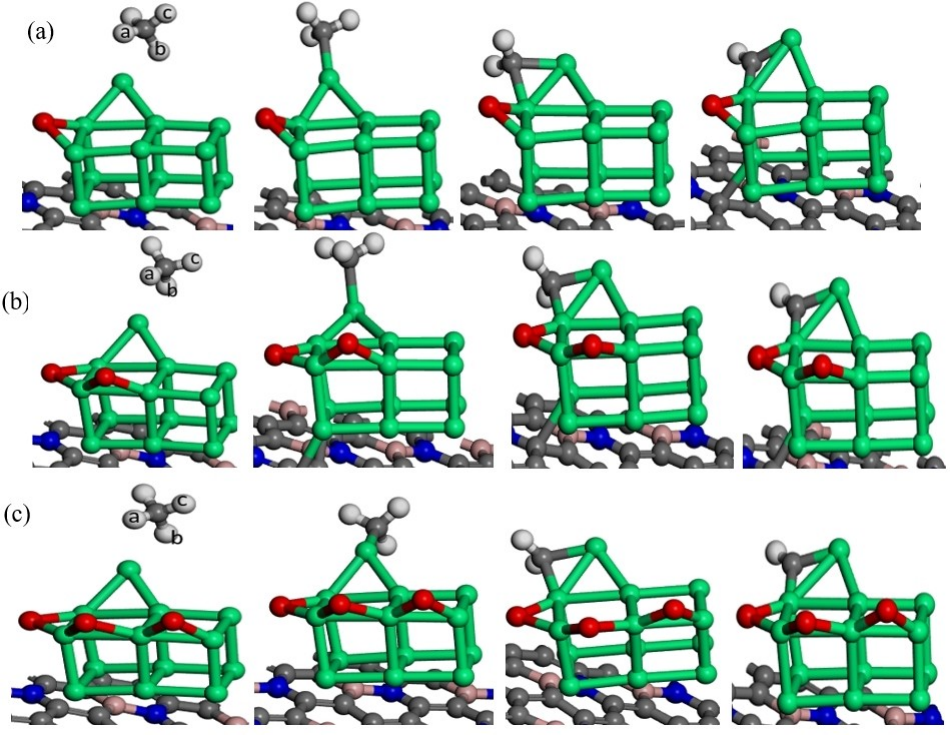
Optimized geometries of the most stable structures of intermediates on (a) BNG‐Ir_13_O cluster, (b) BNG‐Ir_13_O_2_ cluster and (c) BNG‐Ir_13_O_3_ cluster, atomic spheres: green, Ir; gray, C; deep blue, N; pink, B; white, H; red, O.

In order to determine the optimal adsorption structures of methane, we have investigated the most stable geometric structures and adsorption energies of methane by varying the oxygen coverage on BNG‐Ir_13_ cluster. The most stable optimized structures and the adsorption energies of methane including the geometric parameters are shown in Figure 2 and Table 1, respectively. After considering possible configuration of methane in all surfaces, the top site of Ir in oxygen pre‐covered BNG‐Ir_13_ cluster has been found to be the favorable adsorption site of methane. Methane adsorption energies on the BNG‐Ir_13_O, BNG‐Ir_13_O_2_, and BNG‐Ir_13_O_3_ clusters are −0.44 eV, −0.39 eV, and −0.36 eV, respectively. Compared to other intermediates (Table 1), methane has a lower adsorption energy due to its saturated nature and the greater distance between the molecule and the surface. When adsorbed onto the BNG‐Ir_13_O cluster, the hydrogen in the C−H bond of methane pointing towards the surface becomes elongated to 1.13 Å, while on other surfaces such as BNG‐Ir_13_O_2_ and BNG‐Ir_13_O_3_, it elongates to 1.12 Å, suggesting that dissociation of CH_4_ into CH_3_ and H is more likely. The result shows that methane‘s adsorption energy decreases as oxygen co‐adsorption increases, which is in agreement with previous theoretical and experimental results.[^46^, ^47^, ^48^]

**Table 1:** Adsorption energies (*E*_ads_, eV), C−H bond lengths (*d*_C‐Ha,Hb,Hc_, Å), and Ir–C bond lengths (*d*_Ir‐C_, Å) of CH_*x*_ (*x*=0–4) on oxygen pre‐covered BNG‐Ir_13_ cluster. The energy values in parentheses are calculated on BNG‐Ir_13_ cluster.

Species	*E*_ads_ (eV)	*d*_C‐Ha,Hb,Hc_ (Å)	*d*_Ir‐C_ (Å)
BNG‐Ir_13_O cluster			
CH_4_	−0.44 (−0.45)	1.13, 1.12, 1.10	2.45
CH_3_	−2.71 (−2.90)	1.10	2.04
CH_2_	−4.43 (−4.71)	1.10	1.97, 2.05
CH	−6.29 (−6.41)	1.10	1.86, 1.89
BNG‐Ir_13_O_2_ cluster			
CH_4_	−0.39	1.12, 1.12, 1.10	2.54
CH_3_	−3.06	1.10	2.00
CH_2_	−4.67	1.10	1.97, 2.05
CH	−6.42	1.10	1.85, 1.91
BNG‐Ir_13_O_3_ cluster			
CH_4_	−0.36	1.12, 1.12, 1.10	2.53
CH_3_	−2.83	1.10	2.01
CH_2_	−4.52	1.10	1.97, 2.06
CH	−6.53	1.10	1.85, 1.90

The adsorption energy of methane is higher at low oxygen coverage of the BNG‐Ir_13_ cluster and it decreases as oxygen co‐adsorption increases due to the lateral adsorbate repulsion. The oxygen‐pre‐covered BNG‐Ir_13_ cluster suppresses the adsorption energy of methane compared to methane adsorption on the BNG‐Ir_13_ cluster (Table 1). However, when the oxygen coverage increases on the BNG‐Ir_13_ cluster, the adsorption energy of unsaturated intermediates (contrary to that of saturated methane) increases.

**Figure 3:**
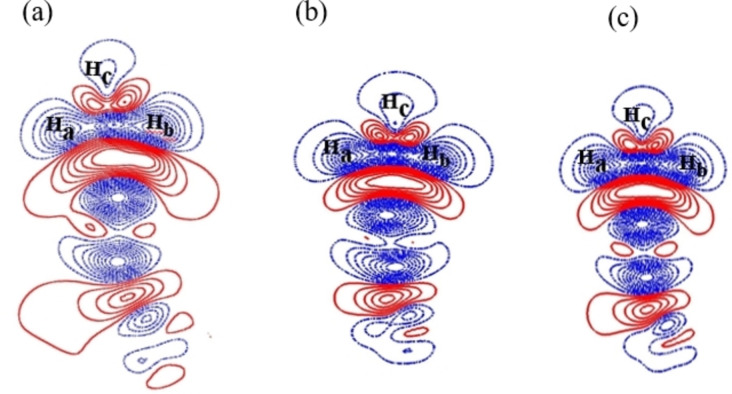
Electron density difference contour plots of CH_4_ on (a) BNG‐Ir_13_O cluster; (b) BNG‐Ir_13_O_2_ cluster; and (c) BNG‐Ir_13_O_3_ cluster, the solid red and dashed blue lines represent increasing and decreasing electron densities, respectively, Iso‐surface value: 0.001

Moreover, the bond length of Ir−C (2.45 Å) in the BNG‐Ir_13_O cluster is shorter than that of the BNG‐Ir_13_O_2_ cluster and BNG‐Ir_13_O_3_ cluster, confirming that the strong interaction of methane and the surface and inconsistent with the higher adsorption energy of methane occurs on low oxygen coverage of BNG‐Ir_13_ cluster than that of high oxygen coverage of BNG‐Ir_13_ cluster. The result is in consistent with the electron density difference (EDD) plot of methane on the surface (Figure 3). It shows that more significant overlapping of orbitals on the BNG‐Ir_13_O cluster occurred more than that of the BNG‐Ir_13_O_2_ cluster and BNG‐Ir_13_O_3_ cluster.

Adsorption of CH_X_ intermediates plays a vital role in methane dehydrogenation, and the hollow site, bridge site, and top site have been considered. We found that Ir′s top site and Ir′s bridge site in the oxygen pre‐covered BNG‐Ir_13_ cluster are the most stable adsorption sites. The adsorption energy of CH_X_ intermediates increases with decreasing number of hydrogen atoms (Table 1), which is in agreement with previous DFT studies.[^49^, ^50^, ^51^] Unsaturated intermediates such as methyl, methylene, and methyne are highly reactive and tend to form new bonds. Table 1 shows the chemisorption energies of CH_x_ intermediates resulting from methane dissociation on an oxygen pre‐covered BNG‐Ir_13_ cluster, while Figure 2 illustrates the corresponding adsorption geometries.

### 2.3 Dissociation of Methane on Oxygen pre‐Covered BNG‐Ir_13_ Cluster

**Figure 4:**
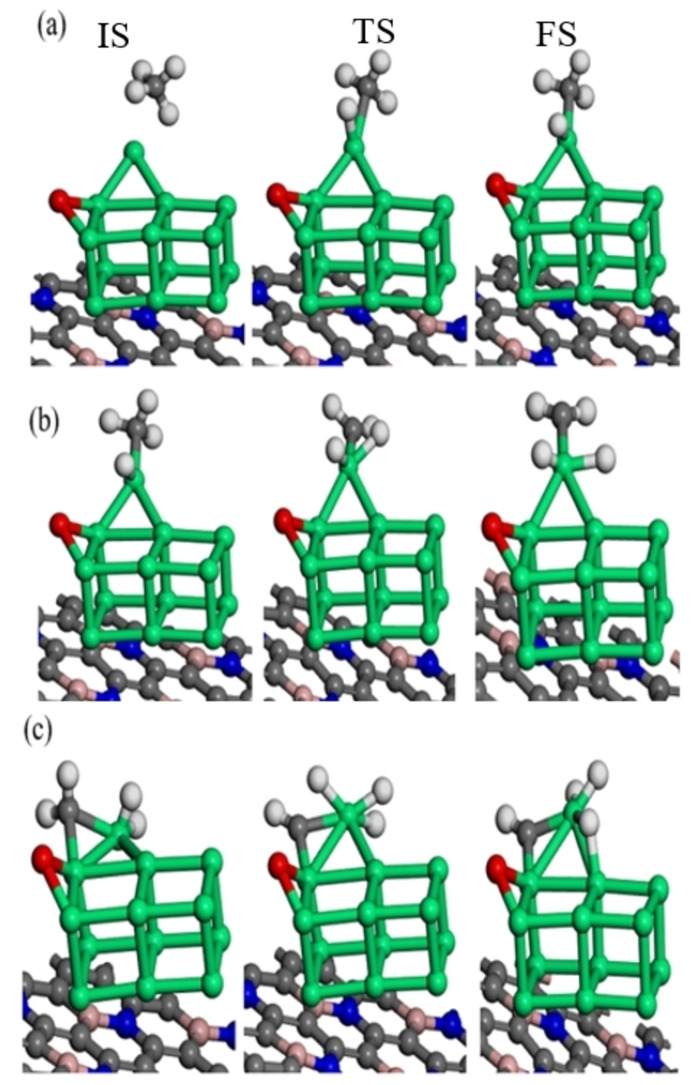
Initial states (IS), transition states (TS) and final states (FS) for dehydrogenation reaction of a) CH_4_, b) CH_3_, and c) CH_2_ on oxygen pre‐covered BNG‐Ir_13_ cluster with low oxygen coverage (BNG‐Ir_13_O cluster), atomic spheres: green, Ir; gray, C; deep blue, N; pink, B; white, H; red, O.

After the most stable adsorption geometries have been determined, methane dissociation occurs through a series of C−H bond cleavage steps, namely CH_4_ to CH_3_ and H, CH_3_ to CH_2_ and H, and CH_2_ to CH and H. This section investigates the dissociation of methane on the BNG‐Ir_13_ cluster under varying oxygen coverage. The initial state is methane‘s stable adsorption site, and the final state is the co‐adsorbed CH_3_ and H. Table 2 provides the reaction energies and activation energies, while Figure 4 and Figure S4 displays the geometric optimization of the initial, transition, and final states. It is apparent that hydrogen, directed toward the surface, undergoes abstraction to produce CH₃ and H species, which reside atop the Ir atoms in an oxygen‐pre‐covered BNG‐Ir₁₃ cluster. The first step of CH_4_ dehydrogenation on the BNG‐Ir_13_O cluster has a 0.24 eV activation energy barrier with an exothermic reaction energy of −0.31 eV. In comparison to the BNG‐Ir_13_O cluster, the methane activation energy barrier increases to 0.39 eV on the BNG‐Ir_13_O_2_ cluster, while the reaction energy remains exothermic at −0.44 eV. To examine the impact of oxygen, the oxygen coverage was increased, and methane‘s dehydrogenation was analyzed. On the BNG‐Ir_13_O_3_ cluster, the initial dehydrogenation stage of methane requires an activation energy barrier of 0.45 eV and results in an exothermic reaction (−0.52 eV). Comparatively, the BNG‐Ir_13_ cluster with high oxygen coverage displays lower methane adsorption energy and higher activation energy barriers for methane activation than the BNG‐Ir_13_ cluster with low oxygen coverage.

**Table 2:** Activation energy barriers (*E*_act_, eV), reaction energies (Δ*E*, eV), activated C−H bond lengths of the transition state (*d*_C−H_, Å) and imaginary frequencies (IMF, cm^−1^) for dehydrogenation reactions in oxygen pre‐covered BNG‐Ir_13_ cluster, and the energy values in parentheses are calculated on BNG‐Ir_13_ cluster.

Reactions	*E*_act_ (eV)	Δ*E* (eV)	*d*_C−H_ (Å)	IMF (cm^−1^)
BNG‐Ir_13_O cluster				
CH_4_→CH_3_+H	0.24 (0.16)	−0.31 (−0.54)	1.43	i783
CH_3_+H→CH_2_+2H	1.24 (1.20)	0.86 (0.92)	1.68	i861
CH_2_+2H→CH+3H	1.03 (0.83)	0.23 (0.30)	1.67	i760
BNG‐Ir_13_O_2_ cluster				
CH_4_→CH_3_+H	0.39	−0.44	1.46	i855
CH_3_+H→CH_2_+2H	1.34	0.76	1.59	i985
CH_2_+2H→CH+3H	1.33	0.59	1.86	i728
BNG‐Ir_13_O_3_ cluster				
CH_4_→CH_3_+H	0.45	−0.52	1.51	i821
CH_3_+H→CH_2_+2H	1.43	0.90	1.62	i931
CH_2_+2H→CH+3H	1.69	0.50	1.66	i803

During the second dehydrogenation step, the dissociation of methyl occurs to yield methylene and hydrogen. This process requires overcoming an activation energy barrier of 1.24 eV, 1.34 eV, and 1.43 eV on the BNG‐Ir_13_O, BNG‐Ir_13_O_2_, and BNG‐Ir_13_O_3_ clusters, respectively. The resulting reaction energies are endothermic with values of 0.86 eV, 0.76 eV, and 0.90 eV on the BNG‐Ir_13_O, BNG‐Ir_13_O_2_, and BNG‐Ir_13_O_3_ clusters, respectively. In this dehydrogenation step, the hydrogen is abstracted and resides on the top site of Ir in oxygen pre‐covered BNG‐Ir_13_ cluster. The methylene intermediate is then rotated to attain stable structures. The second dehydrogenation step is the step that controls the rate of reaction in both the BNG‐Ir_13_O cluster and BNG‐Ir_13_O_2_ cluster. For all surfaces, the activation energy barrier of the second dehydrogenation step increased, making the reaction energy more endothermic compared to the other steps, as shown in Table 2. In the oxygenated BNG‐Ir_13_ cluster, the third dehydrogenation step proceeds by dehydrogenating CH_2_ to CH and H. The activation energy barrier of this step is 1.03 eV, 1.33 eV, and 1.69 eV in the BNG‐Ir_13_O cluster, BNG‐Ir_13_O_2_ cluster, and BNG‐Ir_13_O_3_ cluster, respectively. Although the reaction energy is slightly endothermic in the BNG‐Ir_13_O cluster, it is more endothermic in the BNG‐Ir_13_O_2_ and BNG‐Ir_13_O_3_ clusters. Finally, the third dehydrogenation step controls the reaction rate in the BNG‐Ir_13_O_3_ cluster.

As the oxygen coverage increases in the BNG‐Ir_13_ cluster, the activation energy barriers and reaction energies also increase, except for the first dehydrogenation step. Additionally, the presence of more pre‐adsorbed oxygen on the BNG‐Ir_13_ cluster reduces the interaction between methane and the surface, resulting in decreased catalytic activity compared to the BNG‐Ir_13_O cluster. As presented in Table 2, the BNG‐Ir_13_ cluster demonstrates high activity under oxygen‐deficient conditions. Based on our results, the successive dehydrogenation reaction is as follows: CH_3_>CH_2_>CH_4_ in the BNG‐Ir_13_O and BNG‐Ir_13_O_2_ cluster, whereas CH_2_>CH_3_>CH_4_ in the BNG‐Ir_13_O_3_ cluster. The chemisorbed oxygen with different coverages can alter the activation of methane, which agrees with the previous observations.[^52^, ^53^, ^54^]

### 2.4 Conversion of Methane to C_1_ Oxygenates

**Figure 5:**
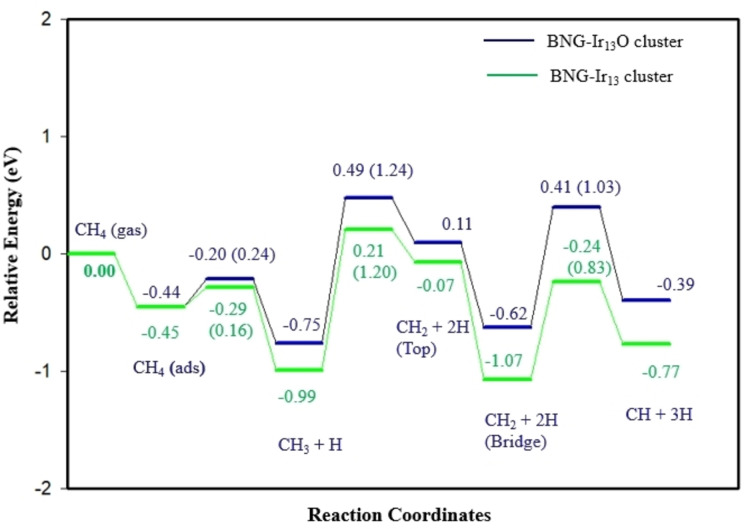
Potential energy diagram for dehydrogenation of CH_4_ to CH and 3H on oxygen pre‐covered BNG‐Ir_13_ cluster and BNG‐Ir_13_ cluster.

**Figure 6:**
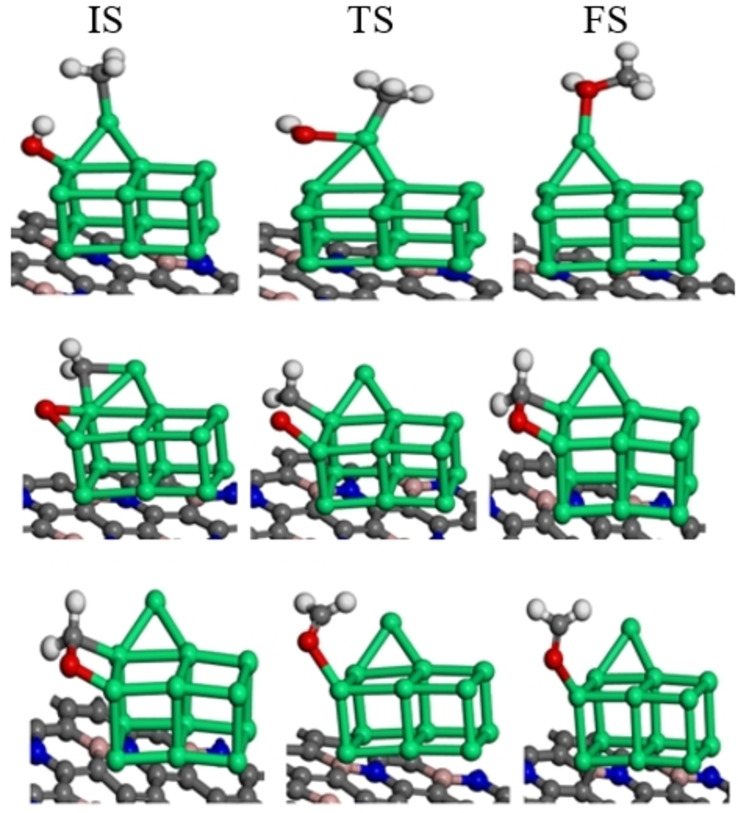
Optimized structures of initial states (IS), transition states (TS) and final states (FS) for C−O coupling reactions on oxygen pre‐covered BNG‐Ir_13_ cluster, atomic spheres: green, Ir; gray, C; deep blue, N; pink, B; white, H; red, O.

Figures 5 shows that the potential energy diagram of methane dehydrogenation reactions in oxygen pre‐covered BNG‐Ir_13_ cluster, where the dehydrogenation of CH_3_ and CH_2_ is kinetically and thermodynamically unfavorable. Thus, the C−O coupling reactions have been studied to form the C_1_ oxygenates by controlling the reaction temperatures and impeding further dehydrogenation of CH_3_ and CH_2_ species. Figure 6 and Figure S5 show the structures of initial states, transition states, and final states of the C−O coupling reactions in the oxygen‐pre‐covered BNG‐Ir_13_ cluster. Table 3 and Table S3 list the activation energy barrier and reaction energy for the formation of methanol on the BNG‐Ir_13_ cluster under low and high oxygen coverage conditions. The results indicate that the activation energy barrier for coupling CH_3_ with a surface oxygen atom is lower in the BNG‐Ir_13_O cluster with low oxygen coverage compared to the BNG‐Ir_13_O_2_ and BNG‐Ir_13_O_3_ clusters with high oxygen coverage (Table S3). When CH_3_OH binds to the surface through the oxygen atom, the C−O axis tilts upward. The reaction energy is 0.48 eV, indicating an endothermic reaction, and the activation energy barrier is 1.37 eV. Hence, the formation of methanol is more feasible on the BNG‐Ir_13_O cluster compared to the BNG‐Ir_13_O_2_ and BNG‐Ir_13_O_3_ clusters.

**Table 3:** Activation barriers (*E*_act_, eV), reaction energies (Δ*E*, eV) and imaginary frequencies (IMF, cm^−1^) for C−O coupling reactions on the most promising BNG‐Ir_13_O cluster (see the Table S3 for BNG‐Ir_13_O_2_ and BNG‐Ir_13_O_3_ clusters).

Reactions	*E*_act_ (eV)	Δ*E* (eV)	IMF (cm^−1^)
BNG‐Ir_13_O cluster			
CH_3_+OH→CH_3_OH	1.37	0.48	i544
CH_2_+O→CH_2_O	1.30	−0.32	i466
η2‐(C−O) CH_2_O→η1‐O CH_2_O	1.02	0.83	i381

**Figure 7:**
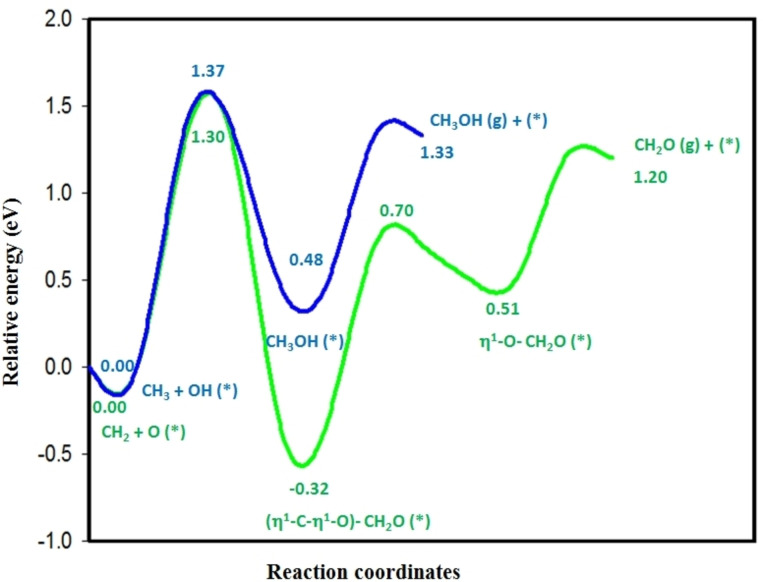
Potential energy diagram for methanol (blue color) and formaldehyde (light green) formation in low oxygen coverage of BNG‐Ir_13_ cluster.

In addition, we examined the formation of formaldehyde on the BNG‐Ir_13_ cluster under low and high oxygen coverage conditions. Our findings reveal that the activation energy barrier for formaldehyde formation is lower (1.30 eV) in the BNG‐Ir_13_O cluster with low oxygen coverage. Moreover, the reaction energy (−0.32 eV) is exothermic, indicating favorable thermodynamic and kinetic conditions compared to the high oxygen coverage of BNG‐Ir_13_ cluster (Table S3). In the final part of our study, we investigated desorption of formaldehyde in the BNG‐Ir_13_O cluster. Our results, presented in Figure 7, show that formaldehyde binds to the surface via the η1–η1 (C, O) configuration, causing the Ir−C bond to break and transfer to the η1 (O) configuration, which binds to the surface through the oxygen atom. The activation energy barrier for this process is 1.02 eV, while the reaction energy is 0.83 eV. The desorption energy of formaldehyde is 0.69 eV, which is lower than that of CH_2_ dehydrogenation. The coupling reactions of CH_3_ and CH_2_ with surface oxygen atoms compete with CH_3_ and CH_2_ dehydrogenation reactions. Therefore, by controlling the reaction temperature, both methanol and formaldehyde can be formed under moderate conditions on oxygen‐pre‐covered BNG‐Ir_13_ clusters with low oxygen coverage.

## 3 Conclusions

The environmentally friendly conversion of methane to value‐added chemicals on BNG‐Ir_13_ at various oxygen coverages is studied by ab‐initio calculations. The adsorption energy of methane on the BNG‐Ir_13_O cluster, BNG‐Ir_13_O_2_ cluster, and BNG‐Ir_13_O_3_ cluster is −0.44 eV, −0.39 eV, and −0.36 eV, respectively, while the corresponding energy barrier for methane activation 1.24, 1.34 and 1.69 eV, respectively. Thus, both these quantities are lower at a lower oxygen coverage. The second (BNG‐Ir_13_O and BNG‐Ir_13_O_2_) or third (BNG‐Ir_13_O_3_) dehydrogenation step is the rate‐determining one.

CH_3_ and CH_2_ dehydrogenation, as well as their coupling with surface oxygen atoms, follow competitive pathways. Assuming abundant CH_3_ and CH_2_ species due to a proper temperature control preventing further dehydrogenation, we investigated C−O coupling reactions on all surfaces. The activation energy barriers for the formation of methanol (1.37 eV) and formaldehyde (1.30 eV) are once again lower on BNG‐Ir_13_O than on BNG‐Ir_13_O_2_ and BNG‐Ir_13_O_3_. Moreover, we considered hydrogen recombination and confirmed that H_2_ can form on these surfaces. Therefore, the BNG‐Ir_13_ cluster with a low oxygen coverage emerges as a potential catalyst for the selective conversion of methane to methanol and formaldehyde, as well as for hydrogen production.

## Author Contributions

**Jemal Yimer Damte**: Writing – review & editing, Writing – original draft, Investigation, Formal analysis, Data curation, Conceptualization. **Jiri Houska**: Writing – review & editing.

## Conflict of Interests

The authors declare that they have no known competing financial interests or personal relationships that could have appeared to influence the work reported in this paper.

## Supporting information

As a service to our authors and readers, this journal provides supporting information supplied by the authors. Such materials are peer reviewed and may be re‐organized for online delivery, but are not copy‐edited or typeset. Technical support issues arising from supporting information (other than missing files) should be addressed to the authors.

Supporting Information
