# Supplementary material for: Formation of C1 oxygenates by Activation of Methane on B, N Co‐Doped Graphene Surface Decorated by Oxygen Pre‐Covered Ir13 Cluster: A First Principles Study
Source: ChemistryOpen. 2025 Feb 11;14(5):e202400287. doi: 10.1002/open.202400287 (PMC12075093; doi:10.1002/open.202400287)
Supplement: Supplementary file 1 — Supporting Information [file OPEN-14-e202400287-s001.pdf]

ChemistryOpen

Supporting Information

Formation of C_1 oxygenates by Activation of Methane on B, N Co-Doped Graphene Surface Decorated by Oxygen Pre-Covered Ir_{13} Cluster: A First Principles Study

Jemal Yimer Damte* and Jiri Houska

Formation of C_1 oxygenates by Activation of Methane on B, N Co-doped Graphene Surface Decorated by Oxygen Pre-covered Ir_{13} Cluster: A First Principles Study

Jemal Yimer Damte*, Jiri Houska

Department of Physics and NTIS – European Centre of Excellence,
University of West Bohemia in Pilsen, Univerzitni 8, 30614 Plzen, Czech Republic

*Email: *damtejem@ntis.zcu.cz*

Adsorption and Activation of methane on high oxygen coverage

The sequential dehydrogenation of methane was investigated on the BNG-$Ir_{13}O_2$ and BNG-$Ir_{13}O_3$ clusters, considering intermediates at the most stable adsorption sites. The results and the optimized geometries of the intermediates are presented in Table 2 and Figure S4. The first dehydrogenation step proceeds with a low activation energy barrier of 0.39 eV on the BNG-$Ir_{13}O_2$ cluster and 0.45 eV on the BNG-$Ir_{13}O_3$ cluster. This process is exothermic with a reaction energy of -0.44 eV on BNG-$Ir_{13}O_2$ and -0.52 eV on BNG-$Ir_{13}O_3$. The second dehydrogenation step exhibits activation energy barriers of 1.34 eV and 1.43 eV for BNG-$Ir_{13}O_2$ and BNG-$Ir_{13}O_3$, respectively, with reaction energies of 0.76 eV and 0.90 eV. The third dehydrogenation step continues with activation energy barriers of 1.33 eV on BNG-$Ir_{13}O_2$ and 1.69 eV on BNG-$Ir_{13}O_3$, both being thermodynamically unfavorable due to endothermic reaction energies. The rate-determining steps are the second and third dehydrogenation steps for BNG-$Ir_{13}O_2$ and BNG-$Ir_{13}O_3$, respectively. The dehydrogenation trend follows $CH_3 > CH_2 > CH_4$ on BNG-$Ir_{13}O_2$ and $CH_2 > CH_3 > CH_4$ on BNG-$Ir_{13}O_3$. By controlling the reaction temperature, C–O coupling reactions were also explored on both clusters.

After activating methane via a low activation energy barrier, the adsorbed methyl species can either undergo further dehydrogenation or couple with other adsorbed species to form C_1 oxygenates. The main strategy for methane conversion involves activating methane at low temperatures while facilitating coupling reactions and inhibiting complete dehydrogenation. C–O coupling reactions, driven by the dominant CH_3 and CH_2 species on both clusters, were also

investigated. The geometric structures of initial, transition, and final states, along with their calculated energies, are shown in Figure S5 and Table S3. The subsequent C–H bond activation in adsorbed methyl species is kinetically unfavorable due to higher activation energy barriers, competing with CH_3/OH and CH_2/OH coupling reactions. These coupling reactions, which are endothermic and kinetically hindered, are unlikely to occur on either cluster. As a result, methane conversion to C_1 oxygenates is challenging on both the BNG-$Ir_{13}O_2$ and BNG-$Ir_{13}O_3$ clusters.

Table S1. Calculated energies (E_{ads}, eV) for Ir_{13} cluster decorated on boron nitrogen co-doped graphene surface.

Possible Structures	E_{ads} (eV) Ir_{13}/BNG
a	-8.38
b	-8.39
c	-8.41
d	-8.12
e	-7.67
f	-7.41
g	-7.61
h	-7.35

Table S2. Activation energy barriers (E_{act}, eV), reaction energies (ΔE, eV) and imaginary frequencies (IMF, cm^{-1}) for dehydrogenation reactions of water on BNG-Ir_{13} cluster.

Reactions	E_{act} (eV)	ΔE (eV)	IMF (cm^{-1})
BNG-Ir_{13} cluster			
$H_2O \rightarrow OH + H$	0.44	-0.82	i1222
$OH + H \rightarrow O + 2H$	1.47	0.46	i1183
$OH + OH \rightarrow O + H_2O$	0.85	0.14	i1275

→

Table S3. Activation barriers (E_{act}, eV), reaction energies (ΔE, eV) and imaginary frequencies (IMF, cm^{-1}) for C-O coupling reactions on oxygen pre-covered BNG Ir_{13} cluster.

Reactions	E_{act} (eV)	ΔE (eV)	IMF (cm^{-1})
BNG-$Ir_{13}O_2$ cluster			
$CH_3 + OH \rightarrow CH_3OH$	1.70	0.85	i474
$CH_2 + O \rightarrow CH_2O$	1.91	-0.12	i451
BNG-$Ir_{13}O_3$ cluster			
$CH_3 + OH \rightarrow CH_3OH$	1.52	0.81	i539
$CH_2 + O \rightarrow CH_2O$	1.98	0.01	i442

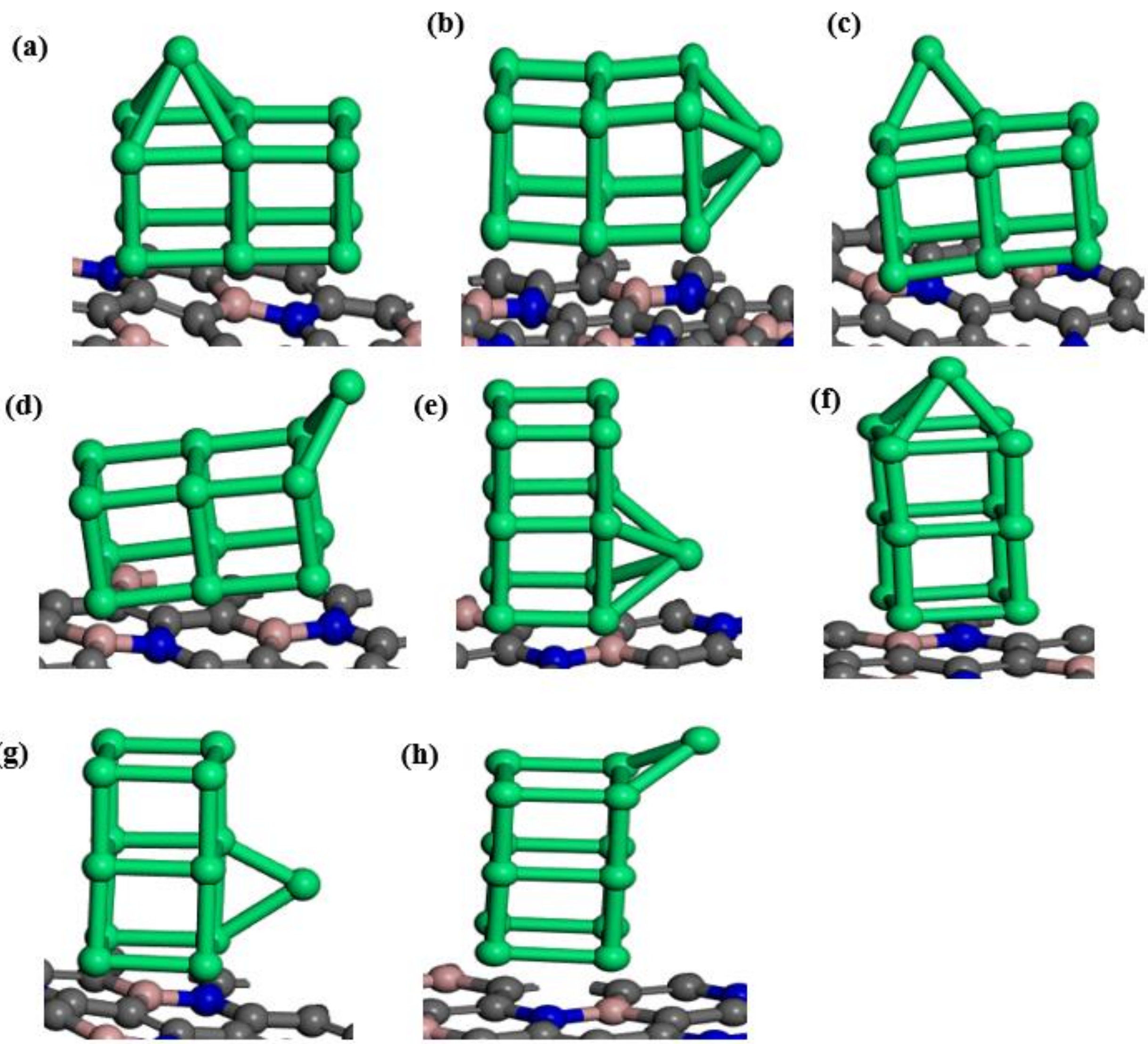

Figure S1 Different possible structures of BNG-Ir_{13} cluster, atomic spheres: green, Ir; gray, C; deep blue, N; pink, B.

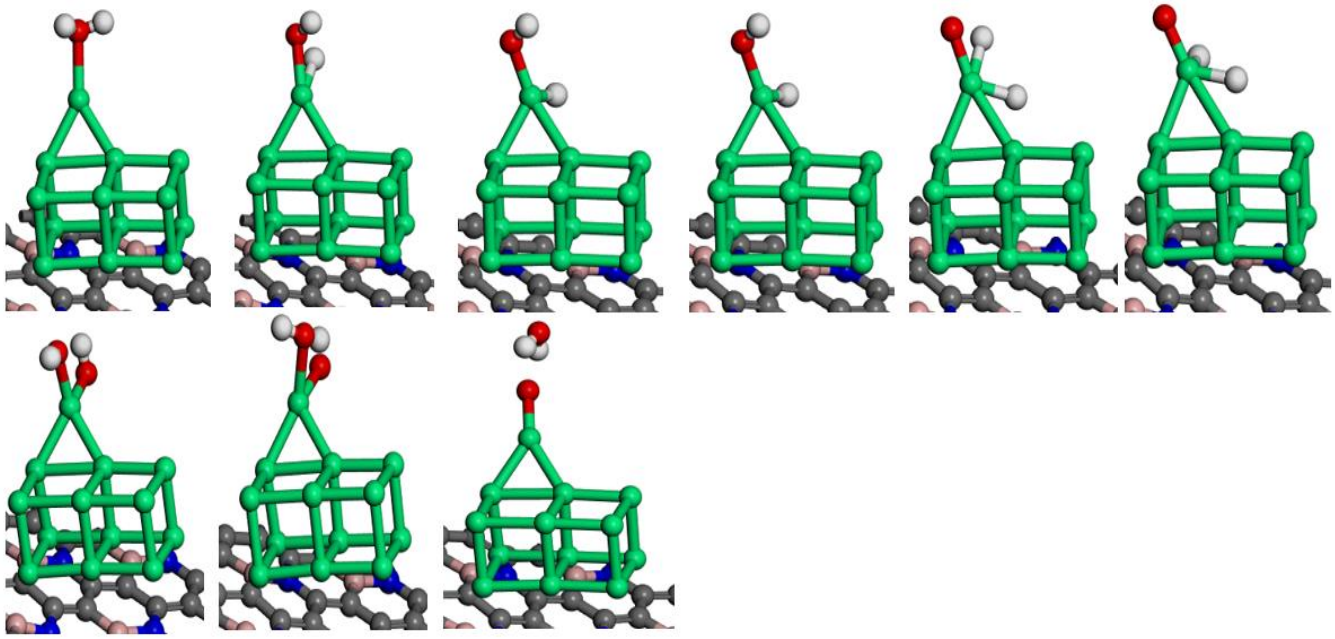

Figure S2. Initial states, transition states and final states for dehydrogenation reaction of water on BNG-Ir_{13} cluster, atomic spheres: green, Ir; gray, C; deep blue, N; pink, B; white, H; red, O.

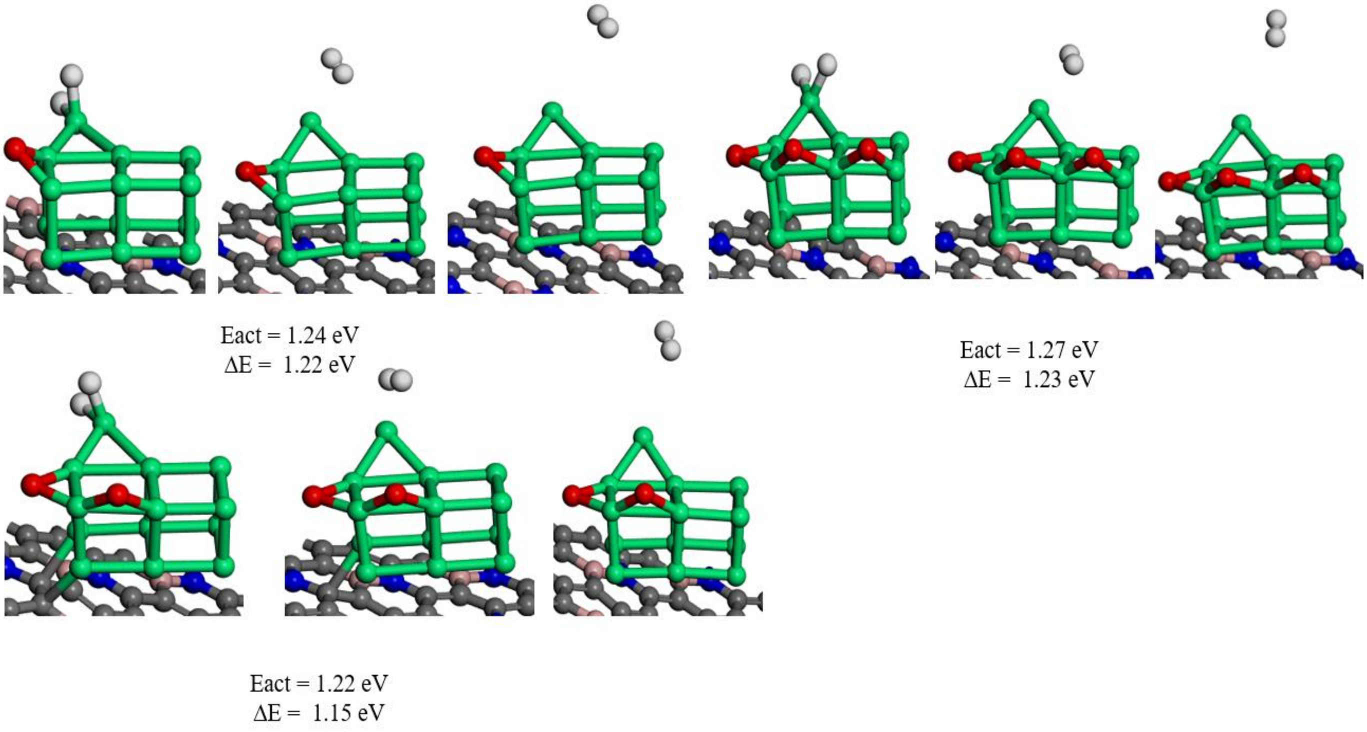

Figure S3. Initial states, transition states and final states of recombination of hydrogen on oxygen pre-covered BNG-Ir_{13} cluster, atomic spheres: green, Ir; gray, C; deep blue, N; pink, B; white, H; red, O.

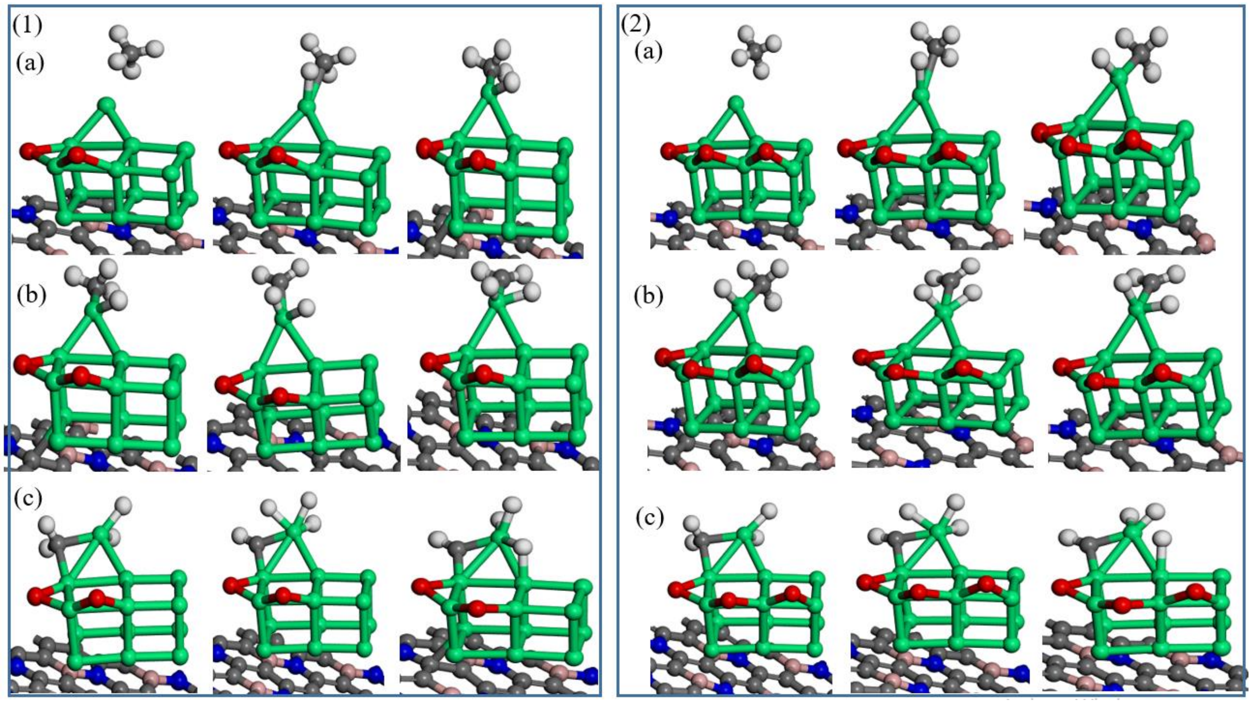

Figure S4 Initial states, transition states and final states for dehydrogenation reaction of a) CH_4, b) CH_3, and c) CH_2 on oxygen pre-covered BNG-Ir_{13} cluster with high oxygen coverage (1) BNG-$Ir_{13}O_2$ cluster and (2) BNG-$Ir_{13}O_3$ cluster, atomic spheres: green, Ir; gray, C; deep blue, N; pink, B; white, H; red, O.

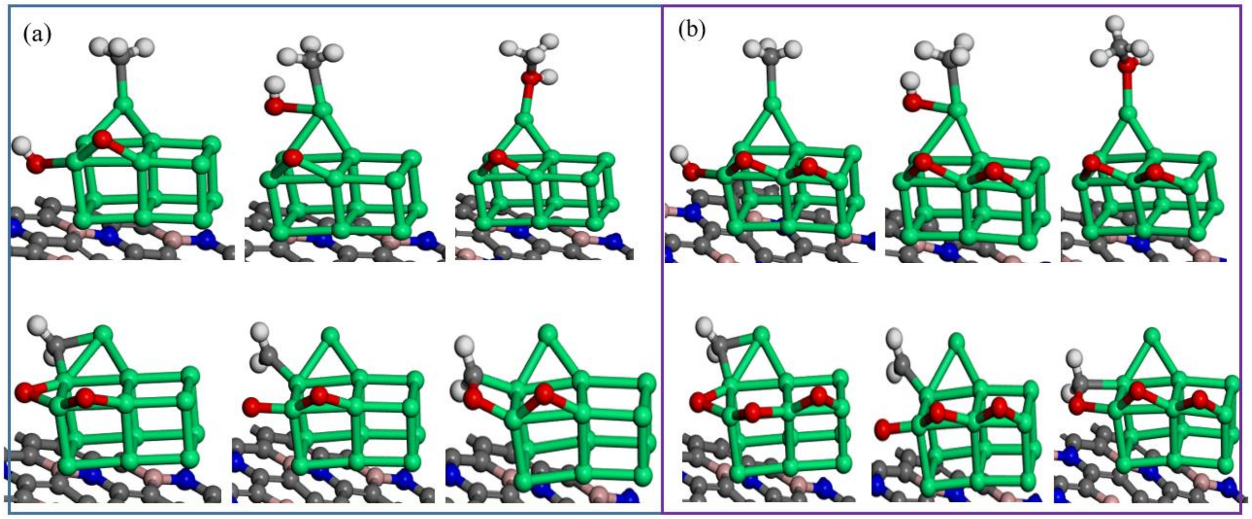

Figure S5. Optimized structures of initial states, transition states and final states for C-O coupling reactions on (a) BNG-$Ir_{13}O_2$ and (b) BNG-$Ir_{13}O_3$ cluster, atomic spheres: green, Ir; gray, C; deep blue, N; pink, B; white, H; red, O.
